# Endoscopic Arthroplasty *via* Mini‐open Direct Anterior Approach Improves Postoperative Complications and Acetabular Components of Total Hip Arthroplasty in Obese Patients

**DOI:** 10.1111/os.14015

**Published:** 2024-02-21

**Authors:** Hanhao Dai, Zhibo Deng, Linhai Yang, Chao Song, Guoyu Yu, Jun Luo, Jie Xu

**Affiliations:** ^1^ Department of Orthopedics Shengli Clinical Medical College of Fujian Medical University Fuzhou China; ^2^ Department of Orthopedics Fujian Provincial Hospital Fuzhou China

**Keywords:** Complications, Direct anterior approach (DAA), Endoscopy, Obesity, Total hip arthroplasty (THA)

## Abstract

To overcome the high‐risk complications and poor alignment of acetabular components in obese patients associated with direct anterior approach (DAA) for total hip arthroplasty (THA), we innovated an endoscopic arthroplasty *via* mini‐open direct anterior approach technique (Endo‐DAA). The purpose of this study was to compare the clinical and radiographic outcomes in obese patients subjected to THA between Endo‐DAA, Bikini DAA, and conventional DAA. In this retrospective controlled study, a total of 360 consecutive primary THA on obese patients (body mass index greater than 28 kg/m^2^) *via* Endo‐DAA, Bikini DAA, and conventional DAA performed from October 2017 to October 2022 by different surgeons and in a single center were included. Assessments including perioperative parameters, clinical outcomes, complications, and radiologic measurements were retrieved from patients before the surgery, perioperative period and the latest follow‐up. A total of 360 consecutive THA (Endo‐DAA = 108, Bikini DAA = 116, Conventional DAA = 136) with complete follow‐up data were analyzed. Compared to Bikini DAA or conventional DAA, Endo‐DAA significantly shortened the length of incision (5.46 ± 0.53), the duration of operation (64.47 ± 12.38), and postoperative hospital stay (2.15 ± 0.89). Endo‐DAA significantly reduces wound related complications compared with conventional DAA. Besides, Endo‐DAA achieved a significantly better alignment of acetabular components compared to Bikini DAA or conventional DAA. Furthermore, Endo‐DAA improved postoperative pain at the activity at 24 h postoperatively and early functional scores. The Endo‐DAA THA technique provides better short‐term clinical and radiographic results in obese patients with a low rate of postoperative complications compared to Bikini DAA or conventional DAA.

## Graphical abstract

- Endo‐DAA improves THA in obese patients.
- Endo‐DAA reduces wound related complications and LFCN injury which are common in obese patients subjected to DAA THA.
- Endo‐DAA has better cup anteversion and inclination and significantly improves the postoperative pain and early function outcomes in obese patients compared with Bikini‐DAA and conventional DAA.

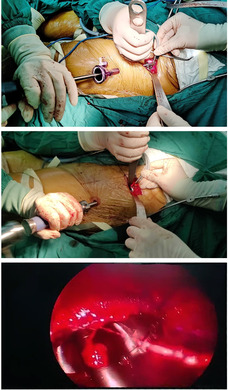

## Introduction

The direct anterior approach (DAA) total hip arthroplasty (THA) exposes the surgical field through the gap between the muscles and has the advantages of rapid recovery, better pain relief, low risk of dislocation, shorter length of stay, early unassisted walking.^1^, ^2^, ^3^ However, the conventional DAA of vertical incision has been reported to be associated with a higher risk of incision infection, delayed wound healing, and reoperation because of its incision location at the inguinal skinfold junction, especially in obese patients.^4^, ^5^, ^6^ As reported, the infection rate of conventional DAA in obese patients ranges between 2.1%^5^ to 4.6%,^4^ while the wound dehiscence rate of conventional DAA in obese patients ranges between 1.96%^7^ to 8.3%.^5^ Barton and Kim.^8^ also found that the vertical incision tended to dehiscence in obese patients with poor skin and overhanging panniculus in the proximal aspect. The Bikini incision technique is a more horizontal skin incision, and placed parallel to the skin creases, which largely corresponds to the skin's tension lines to prevent hypertrophic scarring and optimize cosmetic outcomes.^9^ Though the Bikini DAA of horizontal incision decreases the complication rate mentioned above,^10^ Bikini DAA is still not suited for obese or muscular patients due to the limitation of incision location and size.^11^, ^12^ Leunig *et al*.^11^ pointed out that Bikini DAA was not suitable for obese patients with soft tissues largely overhanging the groin. In addition, Leunig *et al*.^11^ found that the occurrence rate of LFCN dysesthesia rate in conventional DAA and Bikini DAA was as high as 14.0% (7/50) and 28.6% (14/49), respectively, which brought a certain degree of distress to patients.

With the significant increase in the number of obese patients, THA of obese patients has been paid increasing attention by orthopedic surgeons.^13^ Through a literature review, we found that there were still no studies on the application of endoscopy in THA to reduce related complications. To solve the above problems, we innovated an endoscopic arthroplasty *via* mini‐open direct anterior approach (Endo–DAA) for total hip replacement. The Endo‐DAA THA was performed *via* a mini‐open horizontal incision of about 5–6 cm in length and a distal puncture incision approximately 1 cm in length (the details of surgical operation are described in the materials and methods section).

Based on the observation of previous cases, the study found that Endo‐DAA can perform acetabular side operation and femoral side release with good visual field in a small incision, which not only reduces trauma but also has good clinical results. However, there is currently no definitive report on the efficacy and safety of Endo‐DAA in obese patients undergoing THA. The purpose of this study is to: (i) compare the Endo‐DAA technique to the Bikini DAA or conventional DAA in terms of postoperative complications, postoperative complications, radiographic and functional outcomes on obese patients with a body mass index (BMI) of greater than 28 kg/m^2^ ^12^; and (ii) provide better guidance for THA in such patients.

## Materials and Methods

### General Information

This single‐center, multi‐surgeon, retrospective cohort study was performed in line with the principles of the Declaration of Helsinki. All subjects gave their informed consent for inclusion before they participated in the study. The study was conducted in accordance with the Declaration of Helsinki, and the protocol was approved by our Independent Ethics Committee (No: K202‐09‐075).

### Patient Selection

We retrospectively reviewed the clinical records and X‐rays to identify all primary THA performed on obese patients *via* Endo‐DAA, Bikini DAA, or conventional DAA between October 2017 and October 2022.

Inclusion criteria included: (i) the patient had a BMI greater than 28 kg/m^2^; (ii) primary THA; and (iii) the patient was diagnosed with avascular necrosis of the femoral head, femoral neck fracture, congenital hip dysplasia (Crowe type 1, 2), osteoarthritis, rheumatoid arthritis, or ankylosing spondylitis.

Exclusion criteria included: (i) patients with other hip fractures other than the femoral neck; (ii) patients undergoing revision hip arthroplasty; (iii) severe congenital hip dysplasia (Crowe type 3, 4); (iv) combined with serious diseases affecting postoperative rehabilitation exercise, such as severe knee joint disease, spinal disease, etc.; and (v) incomplete follow‐up data of patients. It is worth noting that the retrieval process was completed independently by two scholars. Any inconsistency is resolved through discussion.

**FIGURE 1:**
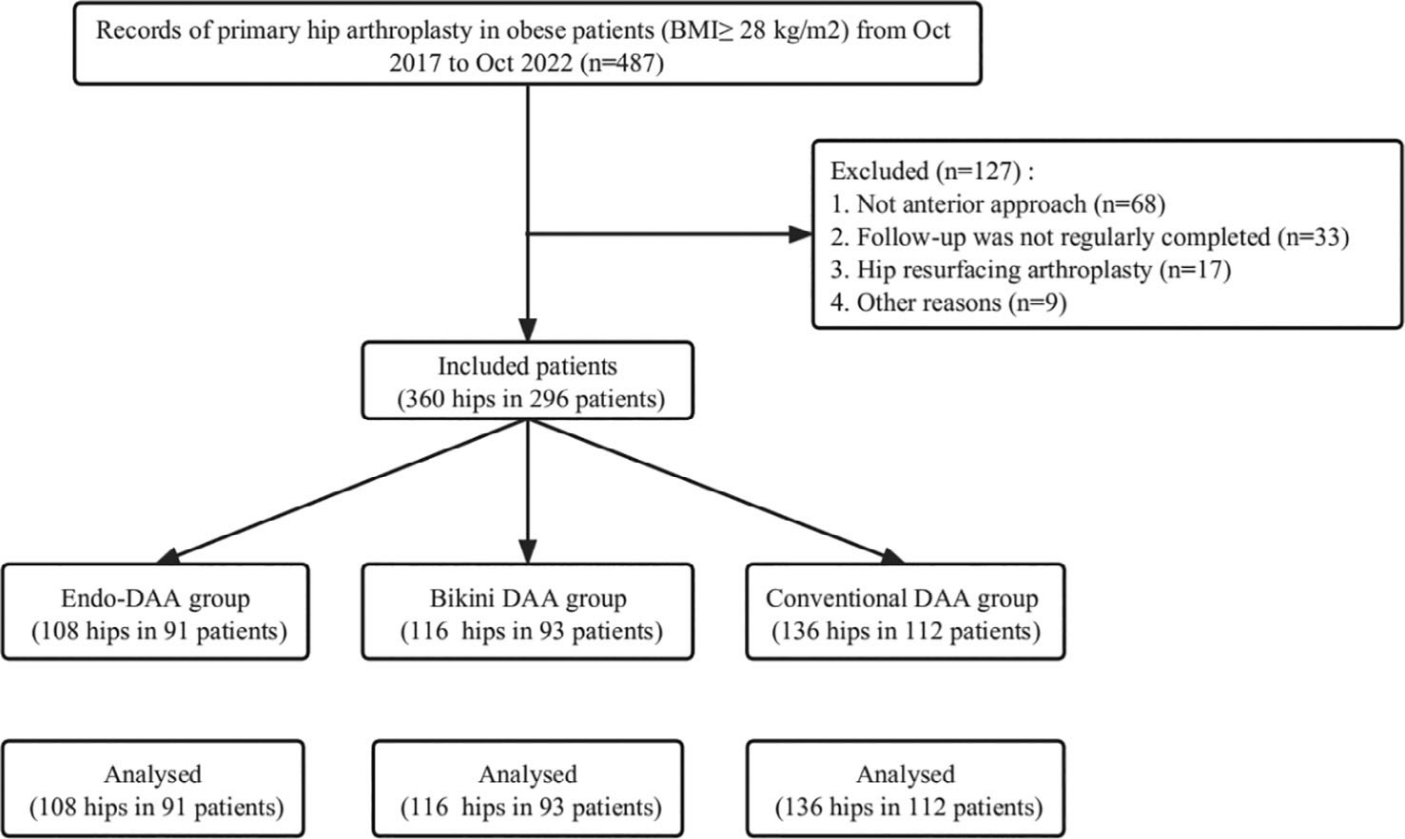
Flow chart of patients included in this study.

A total number of 487 THA with a BMI of greater than 28 kg/m^2^ were recorded. After excluding patients who did not meet the inclusion and exclusion criteria, only 296 patients with 360 hips were included in this study (Figure 1). Among them, 108 THA were performed *via* Endo‐DAA, while 116 and 136 THA was performed *via* Bikini DAA and conventional DAA, respectively. Baseline characteristics including age, gender, BMI, preoperative diagnosis, comorbidities likely to influence postoperative complications, and the patients' physical status according to the American Society of Anesthesiologists (ASA) status^14^ were recorded (Table 1).

**TABLE 1:** Patient baseline characteristics and comorbidities.

	Endo‐DAA (n = 108)	Bikini (n = 116)	Conventional (n = 136)	Statistic values	*p* value
Age (years)	61.34 ± 11.68	61.91 ± 13.34	62.55 ± 12.86	F = 0.277	0.758
Gender				χ^2^ = 1.85	0.396
Female (n)	58 (53.7%)	52 (44.8%)	69 (50.7%)		
Male (n)	50 (47.3%)	64 (55.2%)	67 (49.3%)		
BMI (kg/m^2^)	31.46 ± 2.41	30.98 ± 2.24	31.14 ± 2.25	F = 1.26	0.284
Disease types				χ^2^ = 3.13	0.793
Femoral neck fracture (n)	41 (38.0%)	45 (38.8%)	58 (42.6%)		
Avascular necrosis (n)	35 (32.4%)	38 (32.8%)	40 (29.4%)		
Hip dysplasia (n)	22 (20.4%)	17 (14.7%)	25 (18.4%)		
Osteoarthritis (n)	10 (9.2%)	16 (13.8%)	13 (9.6%)		
Comorbidities				χ^2^ = 13.8	0.180
CHF (n)	1 (0.9%)	5 (4.3%)	2 (1.4%)		
COPD (n)	5 (4.6%)	7 (6.0%)	4 (2.9%)		
Hypertension (n)	28 (25.9%)	32 (27.6%)	39 (28.7%)		
Diabetes (n)	11 (10.2%)	17 (14.7%)	8 (5.9%)		
CHD (n)	7 (6.5%)	3 (2.6%)	8 (5.9%)		
CKD (n)	0 (0.0%)	0 (0.0%)	2 (1.5%)		
ASA status				χ^2^ = 4.52	0.607
1 (n)	4 (3.7%)	8 (6.9%)	9 (6.6%)		
2 (n)	51 (47.2%)	44 (37.9%)	58 (42.6%)		
3 (n)	50 (46.3%)	61 (52.6%)	62 (45.6%)		
4 (n)	3 (2.8%)	3 (2.6%)	7 (5.1%)		

Abbreviations: BMI, body mass index; CHF, chronic cardiac failure; COPD, chronic obstructive pulmonary disease; CHD, coronary heart disease; CKD, chronic kidney disease.

### Surgery and Interventions

#### Anesthesia and Position

**FIGURE 2:**
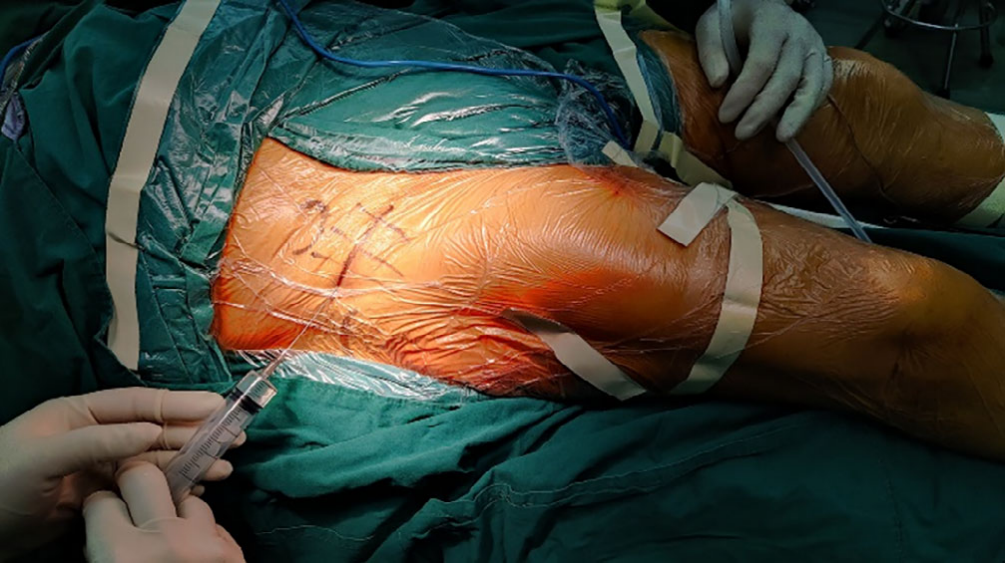
A “cocktail mixture” was locally infiltrating injected before skin incision.

All surgical procedures were performed by different senior surgeons. The same implant was used (Implant: Pinnacle Acetabular Cup System and Corail Hip System by DePuy, Raynham, MA, USA). The patients were placed in the supine position. The pubic symphysis of patients was aligned with the fold of the operating table. Local infiltration analgesia was performed before skin incision (Figure 2).

#### Surgical Procedure

**FIGURE 3:**
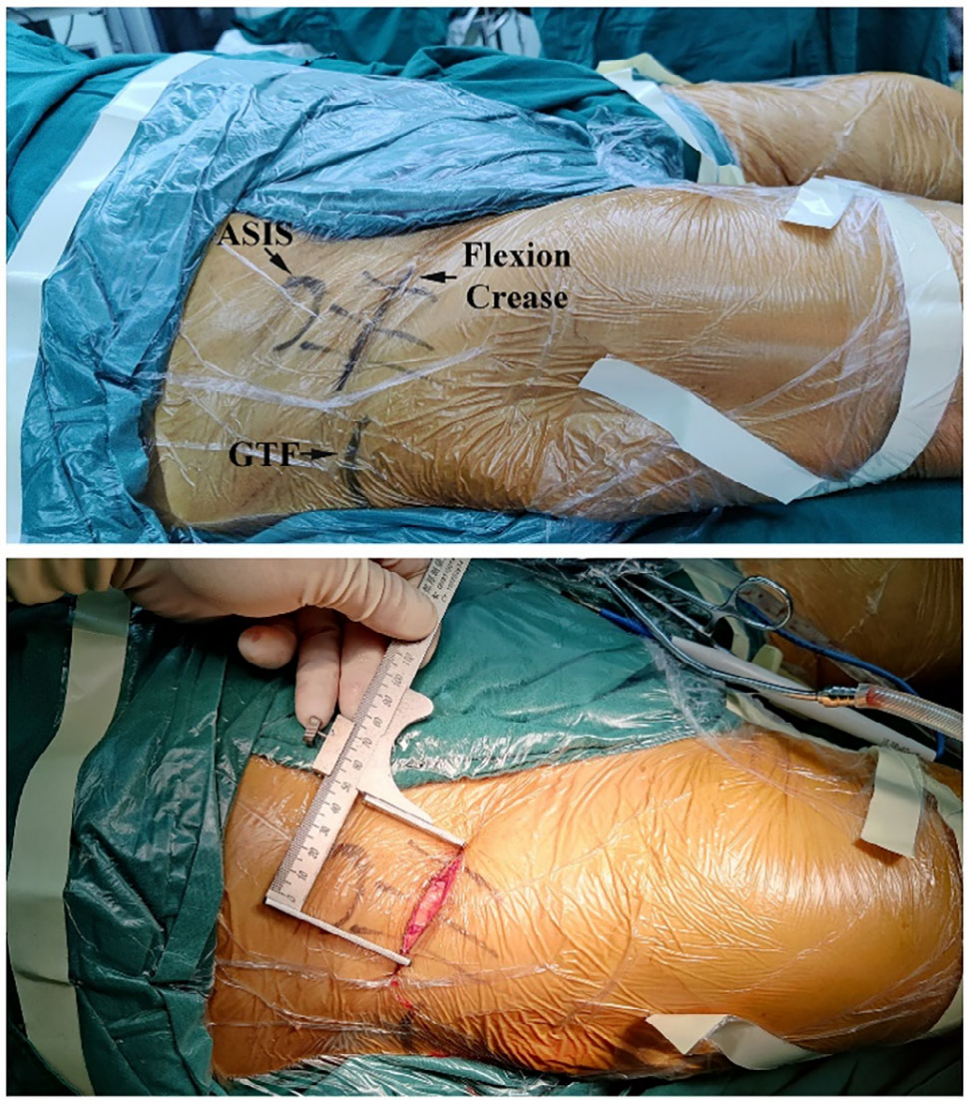
In the Endo‐DAA group, a horizontal incision about 5–6 cm in length was made at the groin crease. ASIS, anterior superior iliac spine. GTF, greater trochanter of femur.

**FIGURE 4:**
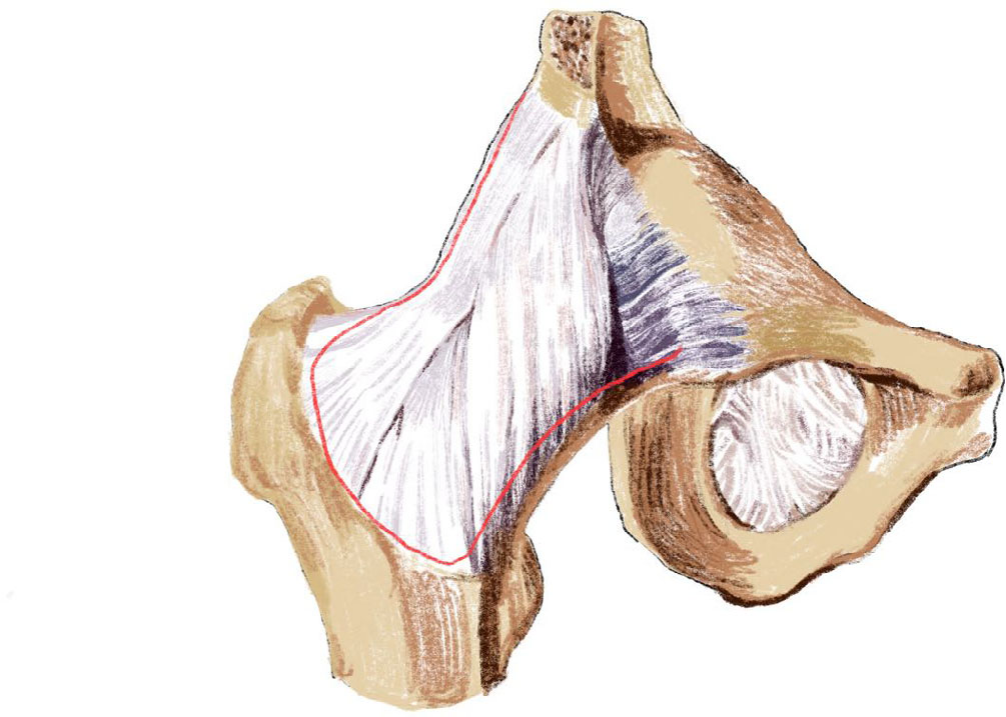
The diagram of incision of the articular capsule. The articular capsule was incised along the red line to form a flap.

**FIGURE 5:**
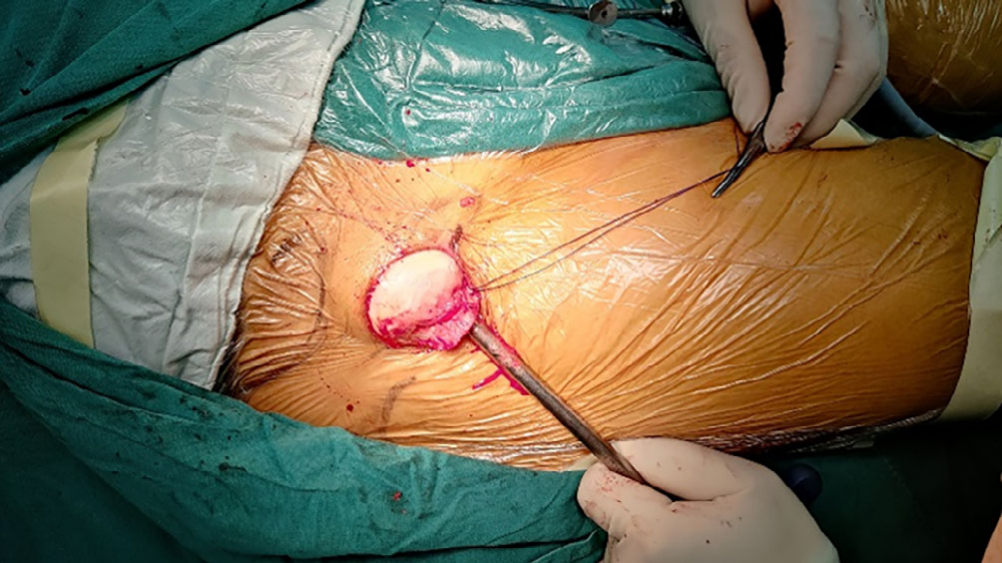
Femoral head was taken out through the minimally invasive incision.

Approach: in the Endo‐DAA group, a 5–6 cm skin incision was placed in the lateral groin crease, and the lateral part of the anterior superior iliac spine should occupy 2/3 of the incision length (Figure 3). To minimize injury to the lateral femoral cutaneous nerve (LFCN), the subcutaneous tissue on the medial side of the Hueter gap was separated and retracted medially. Through the Hueter gap, the lateral femoral circumflex artery was identified, and divided. The articular capsule was incised to form a flap (Figure 4). A Hohmann retractor was used to protect the LFCN. The femoral neck was cut off by a two knife technique to ensure the femoral head was taken out separately through the minimally invasive incision (Figure 5, Video S1).

**FIGURE 6:**
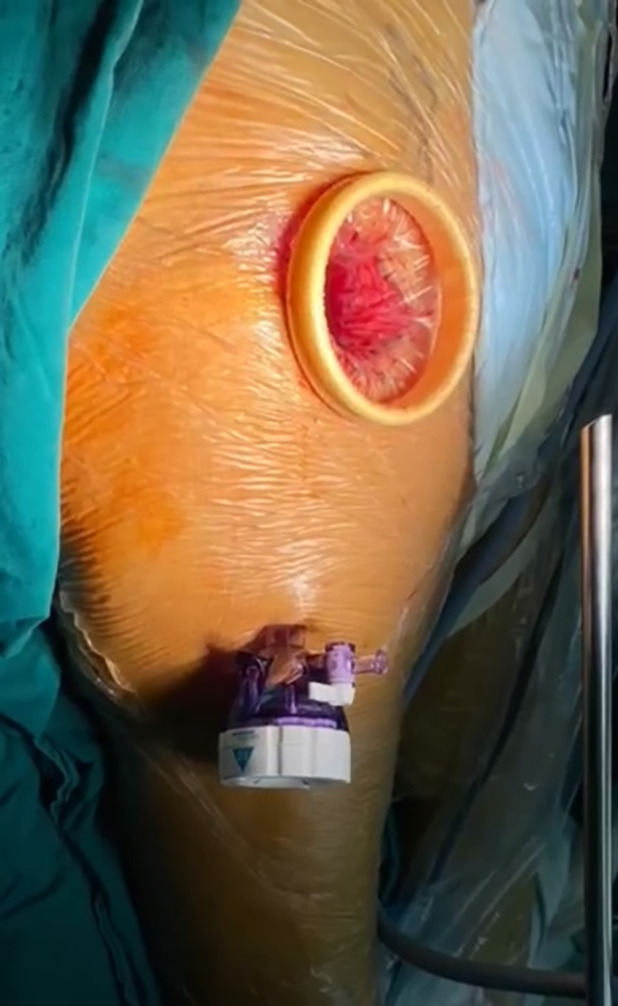
A puncture incision of about 1 cm in length was made about 10 cm away from the horizontal incision distally by using the “finger touch method” through the muscle gap. A trocar with 10 mm in diameter was placed in the puncture incision.

**FIGURE 7:**
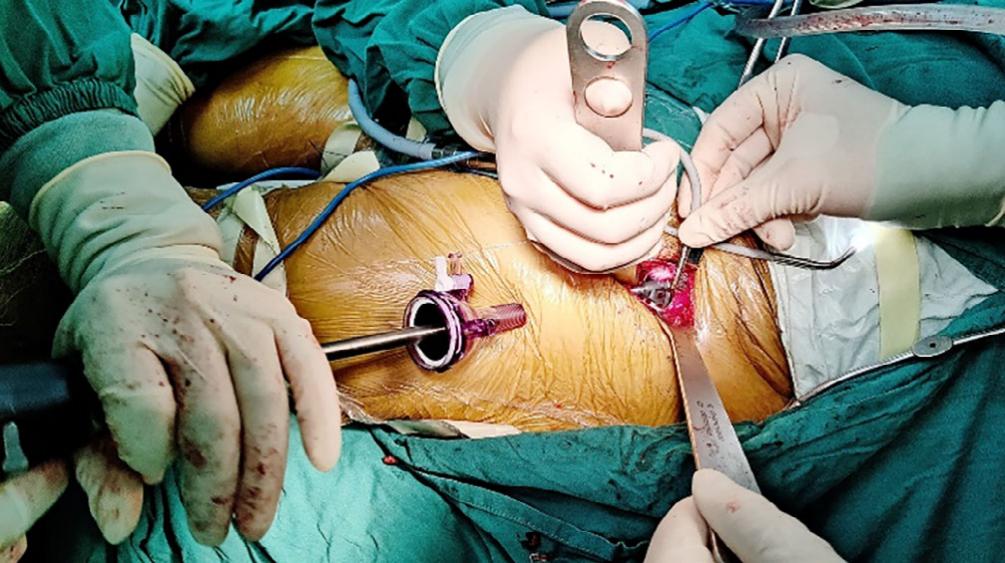
A self‐design split‐type acetabular reamer and handle were placed into the acetabulum through the horizontal incision and the trocar, respectively.

**FIGURE 8:**
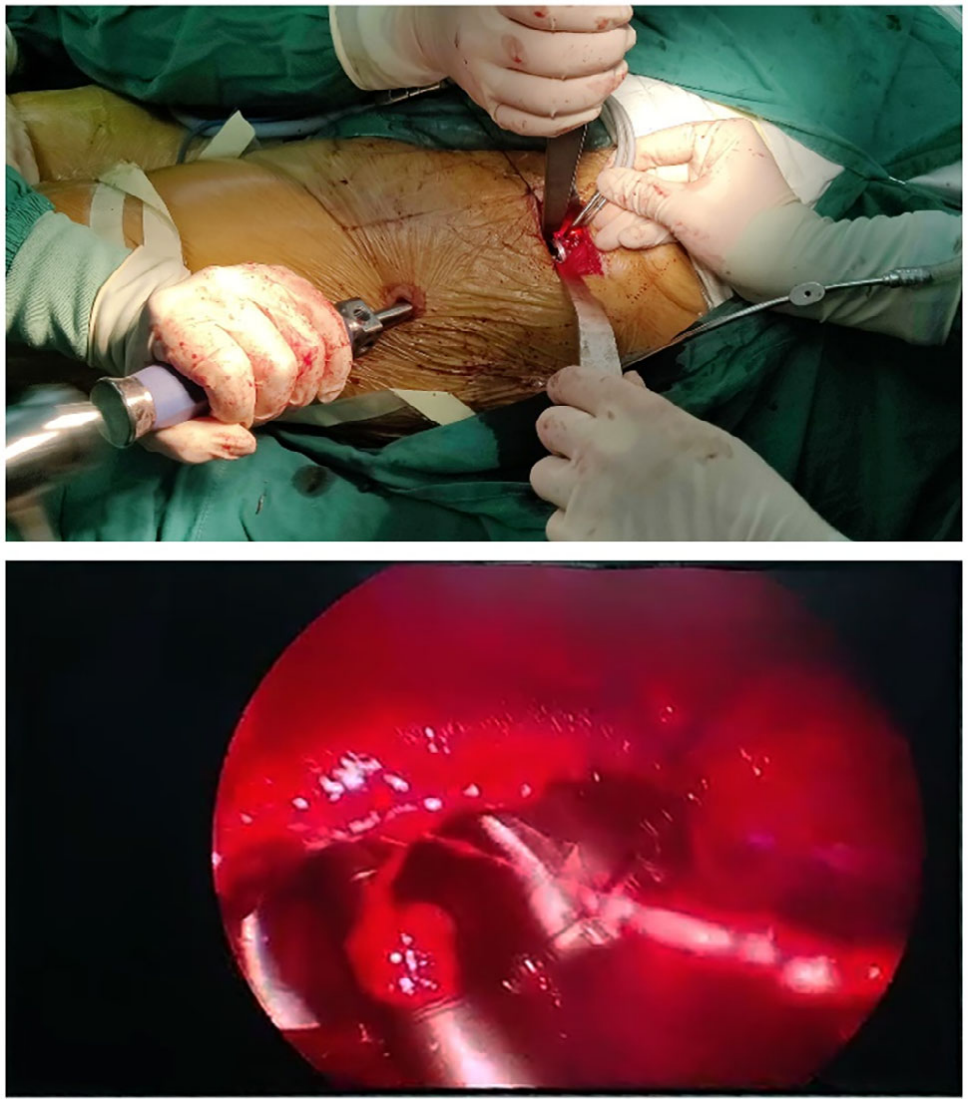
The angles of inclination and anteversion of the acetabular component were adjusted under the endoscopy.

Preparation of the acetabular side: a puncture incision about 1 cm was made and was about 10 cm distal to the surgical incision (Figure 6). Preparation of the acetabulum were performed under the endoscopy (Stryker laparoscope, Kalamazoo, MI, USA). The acetabular reamer and the handle were assembled under endoscopy (Figure 7, Videos S2 and S3). After the prosthesis was properly adjusted, the acetabular prosthesis was pressed through the cannula with a specially designed support rod until it is stabilized (Figure 8, Videos S4 and S5).

**FIGURE 9:**
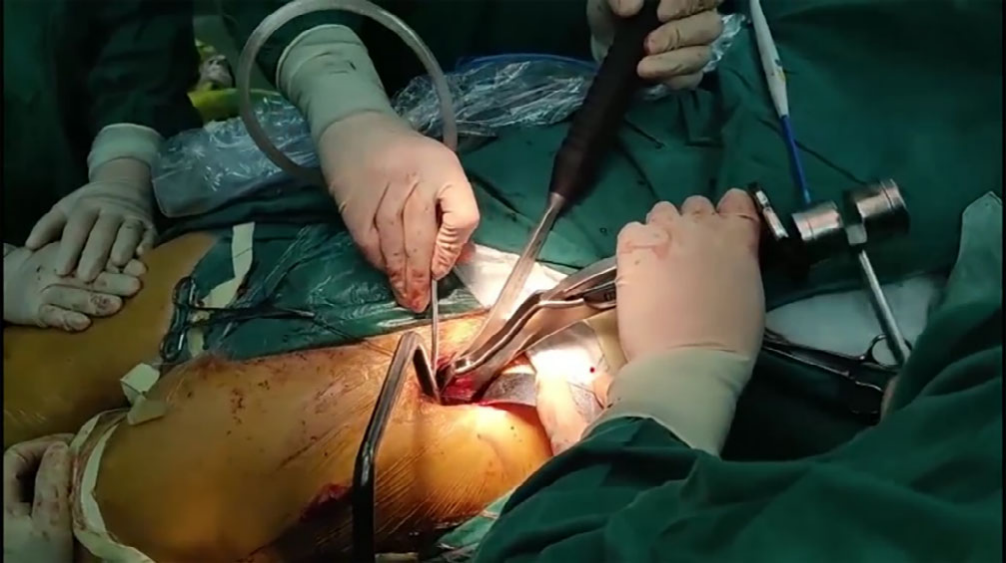
A self‐design lift‐top tractor was placed hooked behind the proximal femur to aid in femoral elevation. Then the medullary cavity was reamed.

Preparation of the femur side: Next, the operating table was bent so that the hip joint was hyperextended by about 30–40°. A lift‐top tractor was hooked behind the proximal femur to aid in femoral elevation. Then the medullary cavity was reamed according to the preoperative measurement (Figure 9, Video S6). After the length of the lower limbs, the stability of the prosthesis, the joint mobility, and the impingement phenomenon were checked, the femoral stem prosthesis and femoral head were inserted.

In the Bikini DAA group and conventional DAA group, the surgical operations were performed through a horizontal incision about 6–8 cm in length^9^ or a vertical incision about 9–13 cm in length at 2 cm distal to the anterior superior iliac spine,^15^ respectively. Neither the endoscopy nor the puncture incision was utilized. In all three groups, surgical incisions were closed by the standard layered manner.

### Perioperative Care

All patients received the same standardized treatment before and after surgery, including pain management and rehabilitation protocols.^16^ In addition, all patients received prophylactic antibiotics and thrombosis prophylaxis.^17^ After full awakening, all the patients were encouraged to walk, progressing from a walker to no assistive devices as could be tolerated.

### Clinical Evaluation

Patients in all three groups were followed‐up clinically and radiographically at 6 weeks, 6 months and 12 months. Scar assessment was performed at the 6‐months follow‐up using the Patient and Observer Scar Assessment Scale (POSAS) and the Vancouver scar assessment scale.^10^ Medical records including operative reports, outpatient clinic notes, and hospital records were collected. Perioperative parameters including length of incision, operation duration, estimated blood loss (EBL), transfusion, and length of stay were recorded. Complications including venous thrombosis, LCFN dysesthesia, surgical site infection, wound dehiscence, hematoma needed revision, periprosthetic joint infection (PJI), periprosthetic fracture, reoperation, and readmission were also recorded. Visual analogue scores (VASs) were used to score pain at rest and activity at 6 h and 24 h postoperatively. Duration to start no‐assistive‐device walking and Harris hip scores (HHSs)^18^ were recorded.

### Radiologic Measurements

**FIGURE 10:**
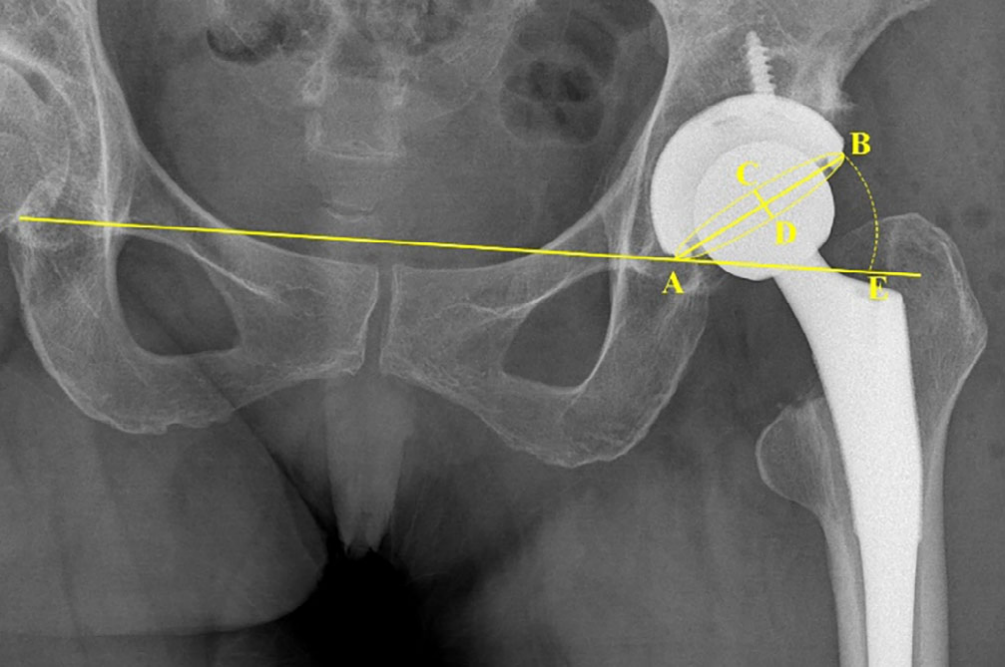
The acetabular component anteversion was calculated using inverse trigonometric functions of the ratio between CD and AB. The degree of ∠BAE was measured as the acetabular component inclination.

The radiological measurement was performed by two orthopedic surgeons blinded to the grouping. On the anteroposterior (AP) pelvic radiograph, anteversion was calculated using the inverse trigonometric functions of the ratio between the lengths of the major and minor axes of the ellipse which was fitted to the rim of the acetabular component, while inclination was measured directly (Figure 10).^19^ The “safe zone” of anteversion and inclination were defined as 5° to 25° and 30° to 50°, respectively.^20^ The leg‐length discrepancy (LLD) was also measured on the AP pelvic radiograph as the perpendicular distance between the corresponding tip of the lesser trochanter to the line passing through both teardrop points.^4^ A positive LLD value indicated that the contralateral side was shorter than the operated limb. Loosening of the acetabular component was defined as a change in the abduction angle of the acetabular component of 5° or migration of 2 mm.^21^

### Statistical Analysis

Statistical analysis was performed with Statistical Package for the Social Sciences (SPSS) version 26.0 (IBM, Armonk, NY, USA). The continuous data conforming to the normal distribution was expressed as mean ± standard deviation (SD). To compare baseline characteristics, surgical variables, and radiologic measurements, the Chi‐square tests or the one‐way analysis of variance (ANOVA) with LSD multiple comparisons tests were used for categorical variables and continuous variables, respectively. Complications were analyzed by using the Fisher's exact test. And two‐way ANOVA with Dunnett's multiple comparisons test was used to analyze VAS. To compare HHS, two‐way ANOVA with Tukey multiple comparisons tests were used. *p* < 0.05 was considered statistically significant.

## Results

### Patients

In the final analysis, there were 91 patients with 108 hips in the Endo‐DAA group, 93 patients with 116 hips in the bikini DAA group, and 112 patients with 136 hips in the conventional DAA group. There were no statistically significant differences in age, gender, BMI, comorbidities, and ASA status between three groups (*p* > 0.05, Table 1). Meantime, there was no statistically significant difference in the proportion of different disease types among the three groups (*p* > 0.05). The highest proportion in each group was the femoral neck fracture, with 41 hips in the Endo‐DAA group and 45 hips in the Bikini DAA group and 58 hips in the Conventional DAA group.

### Perioperative Parameters

**FIGURE 11:**
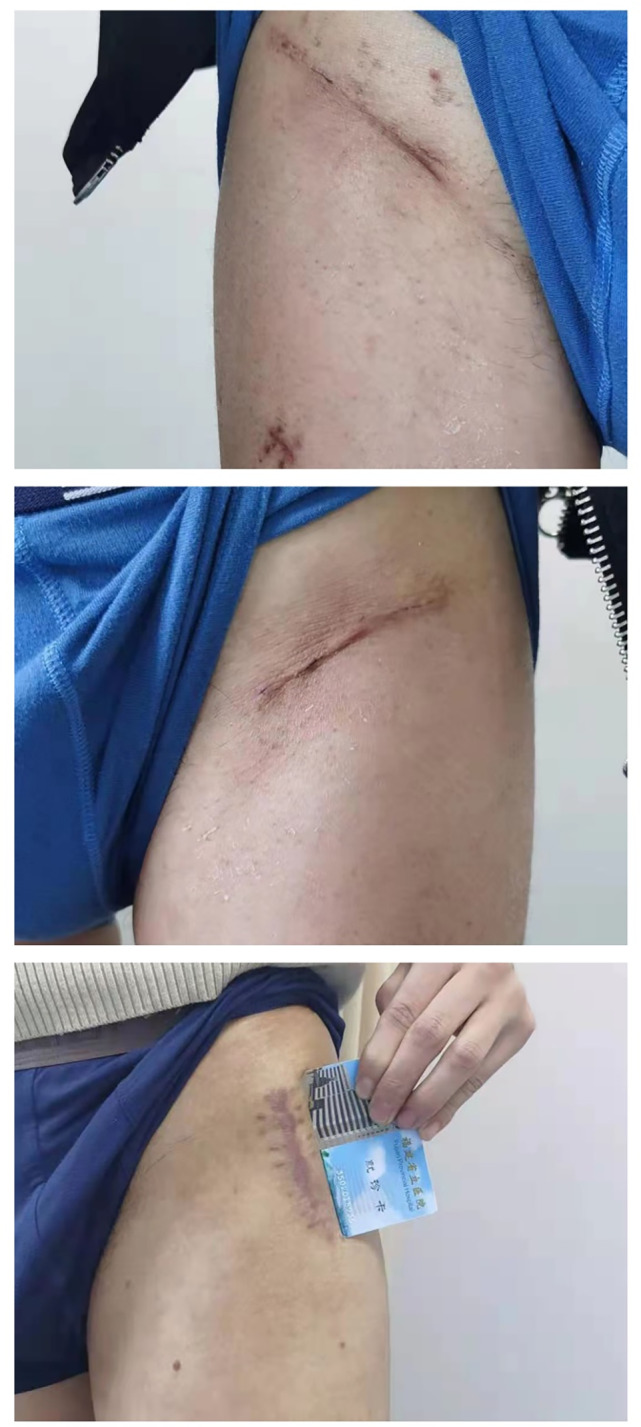
Representative photographs of surgical scars from the Endo‐DAA group (top), the Bikini DAA group (medial), and the conventional DAA group (bottom).

The average length of incision in the Endo‐DAA group was 5.48 ± 0.52 cm, which was significantly shorter than 7.26 ± 1.52 cm in the Bikini DAA group and 8.35 ± 2.03 cm in the conventional DAA group (both *p* < 0.001, Figure 11). Patient reports did not differ in terms of either the POSAS or the Vancouver scale among the three groups. In terms of duration of operation, the conventional DAA was significantly shorter (64.74 ± 12.62 min), followed by Endo‐DAA (65.81 ± 13.45 min) and Bikini‐DAA (96.85 ± 14.03 min). Besides, the postoperative hospital stay in the Endo‐DAA group was 2.23 ± 0.76 days, which was significantly shorter than 4.06 ± 1.08 days in the Bikini DAA group and 4.01 ± 1.18 days in the conventional DAA group (*p* < 0.0001). Besides, the estimated blood loss was significantly less in the Endo‐DAA group (155.41 ± 66.44) than in the Bikini DAA group (176.98 ± 71.17) and conventional DAA group (180.38 ± 78.38) (*p* < 0.01). However, there were no significant differences in the EBL and allogeneic blood transfusion between all groups (Table 2).

**TABLE 2:** Perioperative parameters.

	Endo‐DAA (n = 108)	Bikini (n = 116)	Conventional (n = 136)	Statistic values	*p* value
Length of incision (cm)	5.48 ± 0.52	7.26 ± 1.52^a^	8.35 ± 2.03^b^	F = 104.5	<0.01
POSAS	10.31 ± 2.92	10.43 ± 4.04	10.78 ± 4.32	F = 0.5	0.607
Vancouver scales	5.41 ± 3.78	5.95 ± 4.47	5.96 ± 3.76	F = 0.7	0.497
Duration of operation (min)	65.81 ± 13.45	96.85 ± 14.03^a^	64.74 ± 12.62	F = 221.4	<0.01
EBL (ml)	155.41 ± 66.44	176.98 ± 71.17^a^	180.38 ± 78.38^b^	F = 3.99	0.019
Transfusion (n)	9 (8.3%)	10 (8.6%)	18 (13.2%)	χ^2^ = 2.08	0.354
Postoperative hospital stay (day)	2.23 ± 0.76	4.06 ± 1.08^a^	4.01 ± 1.18^b^	F = 114.3	<0.01

Abbreviation: EBL, estimated blood loss.

^a^ Endo‐DAA *vs*. Bikini DAA group, *p* < 0.05.

^b^ Endo‐DAA *vs*. conventional DAA, *p* < 0.05.

### Postoperative VAS Scores

We found that the Endo‐DAA or Bikini group had a lower active VAS score at 24 h postoperatively than the conventional DAA group (*p* < 0.01) (Table 3). There was no statistical difference among all three groups at other time points (Table 3).

**TABLE 3:** Comparison of VAS scores at rest and activity between all groups at 6 h and 24 h postoperatively.

Postoperative time		Endo‐DAA (n = 108)	Bikini (n = 116)	Conventional (n = 136)	Statistic values	*p* value
6 h	Resting	0.49 ± 0.48	0.53 ± 0.45	0.56 ± 0.47	*F* = 0.677	0.509
	Active	0.74 ± 0.79	0.76 ± 0.80	0.90 ± 0.79	*F* = 1.52	0.219
24 h	Resting	0.89 ± 0.69	1.02 ± 0.78	1.09 ± 0.87	*F* = 1.95	0.144
	Active	1.05 ± 0.76	1.14 ± 0.88	1.46 ± 1.01^a^	*F* = 7.19	<0.01

^a^ Endo‐DAA *vs*. conventional DAA, *p* < 0.05.

### Radiographic Findings

**FIGURE 12:**
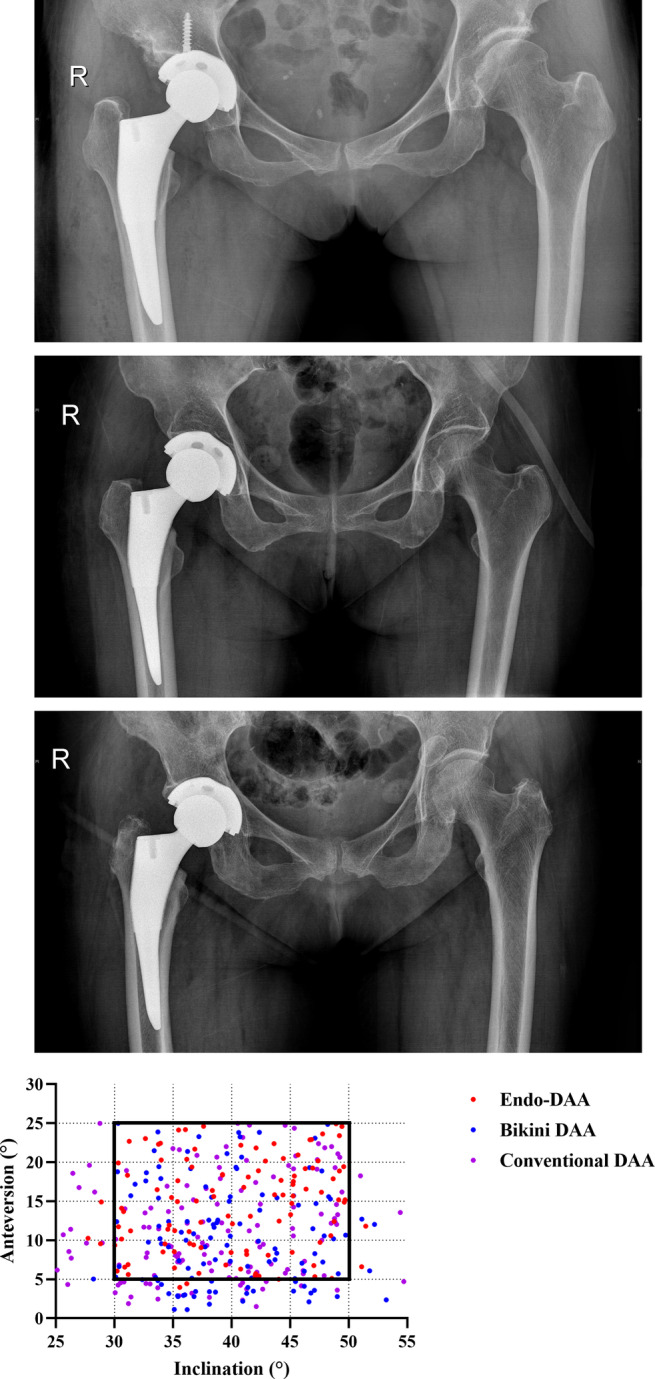
(A) Representative postoperative x‐ray photographs of the Endo‐DAA group (top), the Bikini DAA group (medial), and the conventional DAA group (bottom). (B) Scatterplot of acetabular component alignment in all groups. Square box reflects the “safe zone.”

As shown in Table 4 and Figure 12, the cup anteversion angle of Endo‐DAA group (14.39 ± 5.94°, *p* < 0.05 *vs*. Endo‐DAA and conventional DAA) was significantly better than that in the Bikini DAA group and the conventional DAA group. And the percentage in the “safe zone” of anteversion was higher in the Endo‐DAA group (98.2%, *p* < 0.05 *vs*. Endo‐DAA and conventional DAA) than in the Bikini DAA group and the conventional DAA group. The cup inclination of all three groups was similar (40.10 ± 6.09° in the Endo‐DAA group, 39.89 ± 5.80° in the Bikini DAA group, and 38.99 ± 6.89° in the conventional DAA group) with 95.4%, 95.7%, and 89.0% within the “safe zone” of inclination in the 3 groups, respectively. No significant difference in LLD was observed between all groups. And no loosening of prosthesis was observed in all three groups within the 12‐months follow‐up.

**TABLE 4:** Radiologic measurements.

	Endo‐DAA (n = 108)	Bikini (n = 116)	Conventional (n = 136)	Statistic values	*p* value
Acetabular anteversion (°)	14.39 ± 5.94	10.76 ± 6.53^a^	11.55 ± 6.35^b^	*F* = 10.3	<0.01
Within the “safe zone” (n)	106 (98.2%)	88 (75.9%)^a^	112 (82.4%)^b^	χ^2^ = 22.9	<0.01
Acetabular inclination (°)	40.10 ± 6.09	39.89 ± 5.80	38.99 ± 6.89	*F* = 1.09	0.336
Within the “safe zone” (n)	103 (95.4%)	111 (95.7%)	121 (89.0%)	χ^2^ = 5.65	0.059
LLD (mm)	0.54 ± 4.45	−0.15 ± 5.24	0.23 ± 5.39	*F* = 0.520	0.595
Loosening of acetabular component (n)	0 (0.0%)	0 (0.0%)	0 (0.0%)	NA	NA

Abbreviations: LLD, leg‐length discrepancy.

*Note*: The “safe zone” of acetabular anteversion and inclination were defined as 5° to 25° and 30° to 50°, respectively. A positive LLD value indicated that the contralateral side was shorter than the operated limb. Loosening of the acetabular component was defined as a change in the abduction angle of the acetabular component of 5° or a migration of 2 mm.

^a^ Endo‐DAA *vs*. Bikini DAA group, *p* < 0.05.

^b^ Endo‐DAA *vs*. conventional DAA, *p* < 0.05.

### Functional Outcomes

Of patients in the Endo‐DAA group, 98.1% started no‐assistive‐device walking within 12 h postoperatively. In the Bikini DAA group, no‐assistive‐device walking was started within 12h postoperatively in 39 cases (33.6%), within 24 h postoperatively in 71 cases (61.2%) and over 24 h postoperatively in six cases (5.2%). And in the conventional DAA group, no‐assistive‐device walking was started within 12 h postoperatively in 62 cases (45.6%), within 24 h postoperatively in 47 cases (34.6%) and over 24 h postoperatively in 27 cases (19.9%). The duration to start no‐assistive‐device walking was significantly shorter in the Endo‐DAA group compared to the Bikini DAA group and the conventional DAA group (*p* < 0.01, Table 5). Postoperative HHS scores of every follow‐up improved significantly over time in all groups (*p* < 0.0001). There was no significant difference in the preoperative HHS scores between all groups. The HHS scores at the 6‐week follow‐up in the Endo‐DAA group were statistically higher than that in the Bikini DAA group and the conventional DAA group (*p* < 0.05). No significant difference was observed in the HHS scores at the 6‐month and 12‐month follow‐up between all groups (Table 6).

**TABLE 5:** Comparison of the duration to start no‐assistive‐device walking between all groups.

Postoperative time	Endo‐DAA (n = 108)	Bikini (n = 116)	Conventional (n = 136)	Statistic values	*p* value
<12 h	106 (98.1%)	39 (33.6%)	62 (45.6%)	χ^2^ = 133	<0.01
12–24 h	2 (1.9%)	71 (61.2%)	47 (34.6%)		
>24 h	0 (0.0%)	6 (5.2%)	27 (19.9%)		

**TABLE 6:** Comparison of the preoperative Harris hip score (HHS) and HHS of every follow‐up between all groups.

	Endo‐DAA (n = 108)	Bikini (n = 116)	Conventional (n = 136)	Statistic values	*p* value
Pre‐operation	34.03 ± 6.26	34.29 ± 4.39	34.78 ± 6.57	*F* = 0.523	0.593
6 weeks	79.95 ± 9.21	74.32 ± 9.42^a^	73.52 ± 8.44^b^	*F* = 17.4	<0.01
6 months	91.14 ± 4.85	89.97 ± 5.11	90.25 ± 3.00	*F* = 2.21	0.111
12 months	94.53 ± 3.89	93.81 ± 4.52	94.29 ± 4.37	*F* = 0.831	0.436

^a^ Endo‐DAA *vs*. Bikini DAA group, *p* < 0.05.

^b^ Endo‐DAA *vs*. conventional DAA, *p* < 0.05.

### Complication Rate and Revision

We found that the Venous thrombosis rate and total complication rate was significantly lower in the Endo‐DAA group than in the conventional DAA group (*p* < 0.05). In the Endo‐DAA group, the rates of wound dehiscence and periprosthetic fracture were 0.9% and 0.9%, respectively. The one case of wound dehiscence was treated with disinfection daily for 2 weeks. In the Bikini DAA group, the rates of venous thrombosis, wound dehiscence, and periprosthetic fracture were 1.7%, 2.6% and 0.9%, respectively. Among them, two cases of venous thrombosis were cured after getting out of bed early and combined with oral anticoagulants 4 weeks later; two patients with wound dehiscence were treated with disinfection daily, lasting for 2 weeks, and one case of wound dehiscence was treated with debridement. In the conventional DAA group, the rates of venous thrombosis, LCFN dysesthesia, surgical site infection, hematoma needed revision and periprosthetic fracture were 5.1%, 2.2%, 1.5%, 2.2% and 0.7%, respectively (Table 7). In the conventional DAA group, seven patients with venous thrombosis recovered after getting out of bed early and combining with oral anticoagulants 4 weeks later; three cases of LCFN dysesthesia recovered with oral neurotrophic drugs after 4 weeks; two cases of surgical site infection (present at 7 and 10 days) recovered after treatment with antibiotics based on antibiograms. Also, three cases (2.2%) who required reoperation due to surgical site infection or hematoma improved. The periprosthetic fractures described above were Vancouver type A and recovered after conservative treatment.

**TABLE 7:** Postoperative complications.

	Endo‐DAA (n = 108)	Bikini (n = 116)	Conventional (n = 136)	Statistic values	*p* value
Venous thrombosis (n)	0 (0.0%)	2 (1.7%)	7 (5.1%)^a^	χ^2^ = 6.97	0.031
LCFN dysesthesia (n)	0 (0.0%)	0 (0.0%)	3 (2.2%)	χ^2^ = 4.98	0.083
Surgical site infection (n)	0 (0.0%)	0 (0.0%)	2 (1.5%)	χ^2^ = 3.31	0.191
Wound dehiscence (n)	1 (0.9%)	3 (2.6%)	0 (0.0%)	χ^2^ = 3.86	0.145
Hematoma needed revision (n)	0 (0.0%)	0 (0.0%)	3 (2.2%)	χ^2^ = 4.98	0.083
PJI (n)	0 (0.0%)	0 (0.0%)	0 (0.0%)	NA	NA
Periprosthetic fracture (n)	1 (0.9%)	1 (0.9%)	1 (0.7%)	χ^2^ = 0.028	0.986
Readmission (n)	0 (0.0%)	0 (0.0%)	0 (0.0%)	NA	NA
Total	2 (1.8%)	6 (5.2%)	16 (11.8%)^a^	χ^2^ = 10.1	0.006

Abbreviations: LFCN, lateral femoral cutaneous nerve; PJI, periprosthetic joint infection.

^a^ Endo‐DAA *vs*. conventional DAA, *p* < 0.05.

## Discussion

### Main Findings

The main findings of the present study were that the innovative Endo‐DAA improved the wound‐related complications, alignment of acetabular components, as well as postoperative pain in obese patients compared to Bikini DAA or conventional DAA. Meanwhile, the length of incision, the duration of operation, and postoperative hospital stay of obese patients undergoing DAA THA were shortened by using Endo‐DAA.

### Endo‐DAA Attenuates Wound Related Complication and Postoperative Dysesthesia in Obese Patients

The DAA using a muscle‐sparing technique has obtained popularity for primary THA because of its potential to improve postoperative pain and recovery.^22^, ^23^ However, in terms of complications, mostly infections, dislocation, and wound dehiscence, neither Bikini DAA nor conventional DAA were recommended in obese patients.^4^, ^12^, ^24^ Fortunately, our innovative Endo‐DAA achieved positive clinical and imagological results in obese patients. We conducted this controlled study to explore a relatively better THA surgical approach for obese patients.

According to 6‐month follow‐up, there was no significant difference in scar assessment scales between the three groups, which was consist with the findings of Manrique *et al*.^10^ The most common complications of DAA in obese patients are infection and wound dehiscence required reoperation. We observed three cases (2.2%) in the conventional DAA group subjected to reoperation due to wound complication, which was consistent with the previous studies.^5^ The higher wound complication risk of conventional DAA in obese patients may be related to higher shear forces and proximity to the groin crease. Manrique *et al*.^10^ reported that Bikini DAA was safe for obese patients with no wound‐related complications. In the present study, however, a 2.6% rate of wound dehiscence was observed in the Bikini DAA group. Also, two cases (2.3%) of delayed wound healing were observed in non‐obese patients by Manrique *et al*.^10^ Wang *et al*.^17^ also reported one case (2.0%) of delayed wound healing in non‐obese patients received Bikini DAA THA. A study by Argyrou *et al*. showed that the rate of wound infection in obese patients using traditional DAA was still higher (8.1%). The difference is that we found that Endo‐DAA significantly reduced the risk of wound complications (0.9%).^25^ Di Martino *et al*.^26^ found that obesity patients using traditional DAA showed a higher revision rate (4.87%), and fortunately, Endo‐DAA did not detect any revision during short‐term follow‐up. Therefore, the primary advantage of our innovative Endo‐DAA is to reduce the length of the incision so that the incision can avoid the groin crease. We found that the average length of incision in the Endo‐DAA group was 5.48 cm, which was significantly shorter than that in the Bikini group (7.26 cm) and the conventional DAA group (8.35 cm). The outcomes about length of incision were similar that reported by previous studies.^12^, ^17^ Since Bikini DAA and conventional DAA expose the surgical field and perform surgical procedures through the incision directly, a larger incision is required for better surgical exposure and more ideal alignment of the acetabular component. In Endo‐DAA, the preparation of the acetabular side can be completed by using split‐type tools under endoscopic monitoring. Thus, the incision in the Endo‐DAA is only limited by the size of the acetabular component. And the minimized incision length of Endo‐DAA can decrease the prevalence of wound related complications in obese patients effectively.

LFCN dysesthesia is also a common complication in THA *via* DAA. Goulding *et al*.^27^ reported that up to 88% (53/60) of patients with conventional DAA THA reported LFCN dysesthesia at the early follow‐up. Also 31.9% (39/122) of patients with conventional DAA THA reported LFCN injury in the study of Homma *et al*.^28^ The LFCN has a complex variable branching pattern. And the Bikini DAA incision extends mildly more medially than the conventional DAA incision, which may theoretically increase the risk to LFCN. Although there is no evidence supporting that obesity increases LFCN dysesthesia rates, it is still important to pay attention to LFCN protection. Surprisingly, we only observed three cases (2.2%) of LFCN dysesthesia in the conventional DAA group, while no LFCN dysesthesia was observed in another two groups. One of the reasons may be that we carefully limited the incision length outside the perpendicular line of the anterior superior iliac spine to more than two thirds of the total incision length to protect LFCN from direct trauma. As suggested by Thaler *et al*.^29^ that laterally based incisions over the tensor fasciae latae could minimize the risk to LFCN. In addition, in all three groups, we incised the articular capsule to form a flap and retracted the flap forward. This technique helped us to protect LFCN as described by Zhao *et al*.^30^ Most importantly, it seems that LFCN injury represents a reversible dysesthesia caused by pressure from retractors rather than an irreversible injury caused by dissection. By using Endo‐DAA, we can perform the procedures on the acetabular side under endoscopy without retractors. So, Endo‐DAA can avoid direct and indirect injury to the LCFN in obese patients completely due to its minimally invasive incision and not using retractors.

### Endo‐DAA Improves Cup Anteversion and Inclination in Obese Patients

Another concern about using DAA for THA is reduced intraoperative visualization which may impair the orientation of the components, especially in the Bikini DAA THA. Though several studies reported that cup anteversion and inclination were achieved better in the conventional DAA THA as compared with the posterior approach both with or without fluoroscopy,^19^, ^31^ there were still up to 12.9% (18/139)^19^ acetabular components outside of the “safe zone.” Similarly, Matta *et al*.^32^ found that about 7% acetabular components in the THA using conventional DAA were outside of the “safe zone.” And the rate of the acetabular components outside of the “safe zone” increased to 38% (49/129) in obese patients with conventional DAA THA.^4^ Although no literature reported the rate of the acetabular components outside of the “safe zone” in the Bikini DAA THA, Leunig *et al*.^11^ found that Bikini DAA represented a slightly increased dislocation rate compared to conventional DAA, which may result from an unsatisfied position of the acetabular component. Heinz *et al*.^33^ also showed that obese patients with acetabular protrusion may pose a significant limitation to the DAA. In this study, we found that 98.2% cup anteversion and 95.4% cup inclination in the Endo‐DAA group were inside the “safe zone.” While the rates of the cup anteversion outside of the “safe zone” were 24.1% and 17.7% in the Bikini DAA group and the conventional DAA group with significant differences compared to the Endo‐DAA group. There are two reasons for the excellent acetabular component alignment in the Endo‐DAA. First, all acetabular operations are performed under endoscopy so that we can adjust the direction of the acetabular reamer and the position of the acetabular component intuitively without the obstruction of the visual field by the tools. Second, we grind the acetabulum and press the acetabular component by using the split‐type tools through the puncture incision so that the direction of the tools would not be limited by the size of the incision and the obstruction of the muscles.

### Endo‐DAA Achieves Better Early Functional Results in Obese Patients

In addition, we found that Endo‐DAA improved the VAS scores at activity at 24 h postoperatively and the HHS scores at 6‐week follow‐up compared to the Bikini DAA and the conventional DAA, though the HHS scores at 6‐month follow‐up showed no significant difference between all groups. No venous thrombosis was observed in the Endo‐DAA THA. It may be due to the shorter duration to start no‐assistive‐device walking, fewer wound complications, shorter duration of operation, and better protection of LFCN and muscle in Endo‐DAA THA. Biedermann *et al*. has reported that LFCN dysesthesia resulted in worse functional scores.^34^ Endo‐DAA thus achieves better early functional results. And different from the percutaneously‐assisted THA technique, in which the puncture incision is made from the outside in with the orientation of the locator,^35^ we make the puncture incision from the inside out by using the “finger touch method.” Our puncture incision is made through the muscle gap without injuring the muscle, so that this technique can further protect the muscle in obese patients.

### Limitations and Strengths of this Study

In this study, we reported an innovative Endo‐DAA technique that overcome the disadvantages of Bikini DAA and conventional DAA when be applied to obese patients.

There are precautions and suggestions for the operation of Endo‐DAA. First, Endo‐DAA is not suitable for revision hip arthroplasty. Second, the length of the incision outside the vertical line of the anterior superior iliac spine should occupy two‐thirds of the total length to avoid direct damage to the LFCN. Furthermore, the joint tendons of the superior gemellus muscle, the inferior gemellus muscle and the obturator internal muscles could be clearly seen under endoscopy, and these structures need to be preserved.

In addition, there are still some limitations in the present study. First, because that the Endo‐DAA THA is an innovative technology, the number of patients and the duration of follow‐up are limited. Second, in future studies, more indicators, such as preoperative and postoperative hemoglobin level, serum levels of inflammatory factors and long‐term follow‐up results should be considered.^30^

## Conclusion

In conclusion, our innovative Endo‐DAA improves THA in obese patients. Endo‐DAA reduces wound related complications and LFCN injury which are common in obese patients subjected to DAA THA. Also, Endo‐DAA has better cup anteversion and inclination and significantly improves the postoperative pain and early function outcomes in obese patients compared with Bikini‐DAA and conventional DAA.

## Ethics Statement

The study was conducted in accordance with the Declaration of Helsinki, and the protocol was approved by our Independent Ethics Committee (No: K202‐09‐075).

## Conflict of Interest Statement

The authors declare that they have no competing interests.

## Author Contributions

Hanhao Dai, Zhibo Deng, and Linhai Yang: writing, methodology, investigation; Chao Song: investigation; Guoyu Yu: statistical analysis; Jun Luo and Jie Xu: conceptualization, methodology.

## Supporting information


**Video S1.** Femoral head was taken out through the minimally invasive incision.

**Video S2.** A self‐design split‐type acetabular reamer and handle were placed into the acetabulum through the horizontal incision and the trocar, respectively.

**Video S3.** A self‐design split‐type acetabular reamer and handle were placed into the acetabulum through the horizontal incision and the trocar, respectively.

**Video S4.** The angles of inclination and anteversion of the acetabular component were adjusted under the endoscopy.

**Video S5.** The angles of inclination and anteversion of the acetabular component were adjusted under the endoscopy.

**Video S6.** A self‐design lift‐top tractor was placed hooked behind the proximal femur to aid in femoral elevation. Then the medullary cavity was reamed.
